# CXC Chemokines in the Pathogenesis of Pulmonary Disease and Pharmacological Relevance

**DOI:** 10.1155/2022/4558159

**Published:** 2022-09-17

**Authors:** Kayode Komolafe, Maricica Pacurari

**Affiliations:** ^1^RCMI Center for Health Disparities Research, Jackson State University, Jackson, MS 39217, USA; ^2^Department of Biology, College of Science, Engineering and Technology, Jackson State University, Jackson, MS 39217, USA

## Abstract

Chemokines and their receptors play important roles in the pathophysiology of many diseases by regulating the cellular migration of major inflammatory and immune players. The CXC motif chemokine subfamily is the second largest family, and it is further subdivided into ELR motif CXC (ELR+) and non-ELR motif (ELR-) CXC chemokines, which are effective chemoattractants for neutrophils and lymphocytes/monocytes, respectively. These chemokines and their receptors are expected to have a significant impact on a wide range of lung diseases, many of which have inflammatory or immunological underpinnings. As a result, manipulations of this subfamily of chemokines and their receptors using small molecular agents and other means have been explored for potential therapeutic benefit in the setting of several lung pathologies. Furthermore, encouraging preclinical data has necessitated the progression of a few of these drugs into clinical trials in order to make the most effective use of interventions in the development of viable targeted therapeutics. The current review presents the understanding of the roles of CXC ligands (CXCLs) and their cognate receptors (CXCRs) in the pathogenesis of several lung diseases such as allergic rhinitis, COPD, lung fibrosis, lung cancer, pneumonia, and tuberculosis. The potential therapeutic benefits of pharmacological or other CXCL/CXCR axis manipulations are also discussed.

## 1. Introduction

Chemokines, or cytokines that promote chemokinesis, are a vast family of small, highly conserved proteins that play vital roles in many biological processes, especially in the regulation of cellular migration of immune and inflammatory mediators in the body [1, 2]. This group of small proteins (ranging only from 8 to 12 kDa) is present in all vertebrates and only in some bacteria and viruses, but none is found in invertebrates. Chemokines exist as monomers and dimers, and the monomeric chemokine consists of three *β*-strands, a C-terminal *a*-helix, and an extended N-terminal loop (N-loop) that is critically involved in receptor activation [2]. They have roughly four highly conserved cysteine residues, and this structural trait is used to divide them into subfamilies based on the orientation and proximity of the cysteine to the N terminus [1, 3]. Against this backdrop, the chemokine subfamilies CC, CXC, CX3C, and XC can be distinguished [4]. In the CC chemokines, two of the first four conserved cysteines are adjacent to each other, while there is only a single variable amino acid between them in the CXC chemokines. The CX3C chemokines have three variable amino acids between the two conserved cysteines, but the XC subfamily chemokines lack the first and third cysteines of the motif and have a single variable cysteine residue in the amino terminus [4]. In terms of spread and localization, CC and CXC are the major chemokines, and a large number of their genes have already been characterized [5]. The various functions of chemokines in multicellular organisms, particularly the signature cell migration, are orchestrated through their larger, serpentine G protein-coupled receptors (GPCRs) [6]. Because there are fewer of these GPCRs than chemokine ligands, most receptors bind several ligands [6]. Around 17 of the nearly 50 identified chemokine ligands in humans possess the C-X-C pattern (i.e., CXCL1, CXCL2, CXCL3, CXCL4, CXCL5, CXCL6, CXCL7, CXCL8, CXCL9, CXCL10, CXCL11, CXCL12, CXCL13, CXCL14, CXCL16, and CXCL17). These are ligands for the 9 (out of about 19) identified CXC chemokine receptors, CXCRs (i.e., CXCR1, CXCR2, CXCR3B, CXCR3-alt, CXCR3 (A), CXCR4, CXCR4 variant, CXCR5, CXCR6, and CXCR8) [4, 7]. Chemokines are termed homeostatic or proinflammatory depending on whether they are constitutively produced and function during normal metabolic development or as a result of pathologies arising from or related to inflammatory events, during which time there would be a need to recruit immune cells to the site of infection. The homeostatic CXC motif chemokines responsible for basal leukocyte migration are CXCL 12, CXCL 13, CXCL 21, and CXCL25 [7], whereas the proinflammatory ones include CXCL1, CXCL2, and CXCL8 [8]. Chemokines and their receptors are now known to have important roles in a variety of pathophysiological processes, including inflammation, infectious diseases, allergic responses, autoimmune diseases, and cancer, aside from their involvement in the regulation of leukocyte migration [2, 9]. Because of their direct or indirect involvement in inflammatory and immunological signaling events, this category of cytokines could play a key role in both acute and chronic lung injury and illnesses. There is a paucity of credible appraisals of the roles of CXC motif chemokines and their cognate CGPRs in lung pathologies. The modulation of the CXCR/CXCL axes may have major pharmacological and clinical implications in the context of lung diseases, according to research findings [9]. The current review is thus intended to present the current state of knowledge on this topic in order to provide a solid framework for basic and translational research in this area.

## 2. CXC Chemokine Ligand-Receptor Signalling: A General Overview

The CXC subfamily of chemokines is distinguished by the presence of a variant amino acid residue between the first two conserved cysteines at the amino terminal, otherwise termed the CXC cysteine motif [10]. This chemokine subfamily could be further subdivided depending on whether or not a glu-leu-arg (ELR) amino acid motif that immediately precedes the first cysteine residue is present. The ELR amino acid motif has a significant impact on the characteristics of CXC chemokines. *In vivo and in vitro* chemokines with ELR motif (ELR + CXC) have significant neutrophil chemotactic and activating characteristics [11]. For example, CXCL1, CXCL2, CXCL3, CXCL5, CXCL6, CXCL7 CXCL8, and CXCL15 were discovered to be potent neutrophil chemoattractants in mice following lung inflammation [11, 12]. The CXC chemokines without the ELR motif (ELR^−^ CXC) consist of three members, CXCL9, CXCL10, and CXCL11. These ELR^−^ CXC chemokines exert no chemotactic effects on neutrophils but rather act primarily on mononuclear leukocytes including activated/memory T cells and natural killer cells [10]. CXC chemokines, like other types of chemokines, work by interacting with their corresponding receptors (CXCRs) on the surface of cells, particularly leukocytes [13]. The receptors for these chemoattractants are a family of rhodopsin-like, G protein-coupled receptors (GPCRs) which perform the task of signal transduction via heterotrimeric G proteins [12, 14]. GPCRs are a large family of integral membrane proteins that make up about 3% of the human genome. They are involved in a wide range of physiological activities, including sensory qualities like smell and taste, as well as cellular motility and blood pressure [15]. Responses produced by these receptors are influenced by the pertussis toxin, thus suggesting their coupling to the inhibitory type of G*α* protein (G*α*) [12, 16]. The chemoattractant receptor undergoes a conformational change in response to activation by its agonist (chemokine ligand), which results in the exchange of guanosine diphosphate (GDP) for guanosine triphosphate (GTP) (i.e., a GDP/GTP exchange). Consequently, the G*α*i subunit of the receptor dissociates from the G*βγ* dimer [16] and inhibits adenyl cyclase, an enzyme solely responsible for the synthesis of the second messenger, cyclic adenosine monophosphate (cAMP). The unbound dimer is responsible for the activation of PLC enzymes which catalyze the hydrolysis of the inner membrane component phosphatidylinositol-4,5-bisphosphate (PIP2), into diacylglycerol (DAG) and inositol-1,4,5-triphosphate (IP3). Both DAG and IP3 are second messengers that are vital to the release of intracellular calcium from the endoplasmic reticulum and the activation of protein kinase C and other calcium-sensitive protein kinases to drive major cellular events [14, 16]. Phosphoinositide 3-kinase (PI3K)*γ*, the enzyme responsible for the conversion of phosphatidylinositol 4,5-bisphosphate (PIP2) to phosphatidylinositol (3,4,5)-trisphosphate (PIP3), is also produced by the G*βγ* dimer. G*βγ* also activates other kinases that are under the regulation of extracellular signal, including ERK and protein kinase B (Akt) which is involved in a variety of physiological activities such as apoptosis and cell migration. Coupling and signaling through additional signal mediators like *β*-arrestin may also be involved in the functioning of chemokine receptors, in addition to G proteins [14, 17]. When GPCRs' intracellular C-termini are phosphorylated by both GPCR kinases (GRKs) and second messenger-dependent kinases in response to agonist activation and the accompanying signaling cascades, they become desensitized, albeit rapidly. This event causes the recruitment of *β*-arrestin to the receptor [17]. Furthermore, the activation of other downstream pathways for a particular chemokine-receptor interaction varies. The response elicited by a given chemokine ligand is determined by the chemokine's cellular location as well as the type of cognate receptor to which it binds [14, 18].

## 3. The Role and Therapeutic Relevance of CXC Motif Chemokines and Receptors in Pulmonary Diseases

Chemokines play a role in immune cell modulation in both healthy and pathological situations, such as inflammatory and allergic reactions. As a result, they are predicted to play a critical role in lung illnesses that have both immunological and inflammatory underpinnings. The role of CXC motif chemokine ligands/receptors in the development of pulmonary allergic disorders is explored.

### 3.1. Hypersensitivity Pneumonitis and the CXC Motif Chemokines

Also known as extrinsic allergic alveolitis, hypersensitivity pneumonitis (HP) is a term that describes lung alveoli inflammation commonly caused by occupational exposure to dust [19]. HP is a kind of allergic lung disease that, like other allergy ailments, develops as a result of an overactive immune response to contact with a foreign substance, which is often impacted by genetic susceptibility. The allergic inflammatory disease is of helper T-cell (Th1) origin, and the formation of small areas of inflammation (granulomas) is dependent on the Th1 cytokine, interferon-gamma (IFN-*γ*), [20]. Allergic reactions typically accelerate inflammation cascades, in which inflamed or damaged tissues release inflammatory chemicals, mainly cytokines, in order to recruit immune cells, with varying outcomes. Exposure to *Saccharopolyspora rectivirgula* (SR), a particulate antigen, increased the production of IFN-inducible C-X-C motif chemokines, including CXCL9 (monokine induced by gamma, MIG), CXCL10 (interferon gamma-induced protein 10, IP-10), and CXCL11 (otherwise known as interferon-inducible T-cell alpha chemoattractant). This was accompanied by the recruitment of CXCR3(+)/CD4(+) T cells into the lung there by affirming the role of IFN-*γ* in mobilizing the cognate receptors and in the production of the CXC chemokines. Similar other findings established the induction of CXCR3(+) T-cell line chemotaxis by alveolar macrophages following secretion of CXCL10 in response to IFN-*γ* [21]. CXCR3(+)/IFN(+) cytotoxic T-cell type 1 (Tc1) cell accumulation in the bronchoalveolar lavage (BAL) in hypersensitivity pneumonitis is mediated by IFN-*γ*, which facilitates the expression and secretion of CXCL10, which induces lymphocyte chemotaxis and alveolitis sustenance, according to Agostini et al. [22]. CXC chemokines and/or their corresponding receptors are involved in these IFN-induced actions, which finally lead to Tc1-cell alveolitis and granuloma formation [23].

#### 3.1.1. Therapeutic Potential of CXCRs Antagonism in Hypersensitivity Pneumonitis

Despite indications that the CXCL10/CXCR3 axis is involved in the development of hypersensitivity pneumonitis (HP), nothing is known about the therapeutic benefits of small molecule suppression of the pair components. Knocking out IFN-*γ* was demonstrated to prevent C-X-C motif chemokines mobilization, recruitments of CXCR3(+)/CD4(+) T cells into the lung, and eventual alveolitis and granuloma formation [20, 23]. Andrews et al. [24] reported that antigen-induced hypersensitivity pneumonitis (HP) pathology was accompanied by toll-like receptors (TLRs) 2- and 9-dependent induction/production of granulocyte colony-stimulating factor (G-CSF) that stimulates neutrophil production and recruitment of neutrophil chemokines, CXCL1, CXCL2, and CXCL5, into the airways. In induced animals, a decrease in neutrophil recruitment was positively linked with a decrease in alveolitis [24]. Knocking out TLRs 2 and 9 could decrease levels of some inflammatory cytokines and the extent of neutrophil recruitment into the airways but could not stop or mitigate granuloma formation, thereby suggesting the involvement of additional mediators and players in the disease [24]. However, after *Methanosphaera stadtmanae* (MSS)-induced HP in mice, considerable numbers of B-cell-rich tertiary lymphoid tissues, which are thought to be important for antigen-specific responses and are affected by CXCL13, were massively produced [25]. Treatment of animals with RP001, a small molecular inhibitor of sphingosine-1-phosphate receptor 1 (S1P1), prevented the increase of CXCL13 in the pulmonary tissues, and this was linked to complete inhibition of tertiary lymphoid tissue enlargement and a significant decrease in pulmonary MSS-specific antibody titers [25]. From current indications, CXCL13 and its receptor CXCR5 could be involved in the pathophysiology of HP being an autoimmune-related disorder and therefore deserve full investigation for the possible unveiling of novel effectors and therapeutic opportunities. The strong evidence for the crucial involvement of the CXCR5 : CXCL13 axis in lymphoid cell biology and in facilitating cell-cell interactions that promote lymphocyte infiltration [26] fuels speculation of potentially successful immune-targeted therapies in patients with hypersensitivity pneumonitis.

### 3.2. Allergic Rhinitis and the CXC Motif Chemokines

Allergic rhinitis is an inflammatory allergic reaction in the nose that is caused by an overreaction to allergens in the air. The disorder is closely linked with asthma and a line of evidences has revealed that it involves the whole respiratory tract rather than being only localized to the upper respiratory tract [27]. Pollen, dust, animal furs, and mold are notable environmental triggers of the condition in which symptoms include sneezing, stuffy nose, and usually clear nasal discharge [28]. Allergic rhinitis is immunoglobulin-E (IgE)-mediated and driven by type 2 helper T (Th2) cells. CXC motif chemokines and their receptors are postulated to be vital to this process [28]. CXCL10 or interferon (IFN)-*γ*-induced protein 10 (IP-10) is involved in the targeted migration of cell types associated with inflammation at several levels. These cell types include but are not limited to activated T cells and natural killer cells [29]. CXCL10 is also involved in the induction of Il-8 and CXCL5, promotion of apoptosis, and inhibition of proliferation in several cell types [30]. Levels of circulating CXCL10 are known to increase with age, in some forms of cancer and in some autoimmune diseases [31]. Work by Zhang et al. [32] revealed that single-nucleotide polymorphisms (SNPs) in CXCL9, CXCL10, and CXCL11/IFN-inducible T-cell alpha chemoattractant (I-TAC) are strongly associated with seasonal allergic rhinitis. The concentrations of both CXCL9 and CXCL10 were also found to be elevated in nasal lavages from allergic patients suggesting they might be crucial to chronic allergic inflammation [23, 32].

#### 3.2.1. Therapeutic Potential of CXCRs Antagonism in Allergic Rhinitis

CXCR2 antagonists could offer a probable strategy to prevent allergic airway inflammation in allergic rhinitis caused by pollen exposure. Ragweed pollen extract exposure in mice resulted in neutrophil recruitment into the airways occasioned by CXCL1 and CXCL2 chemokine synthesis and the eventual stimulation of CXCR2-induced recruitment of the inflammatory leukocytes [33]. These inflammatory events were prevented by treatment with SB225002, a CXCR2 antagonist [33]. Also, ketotifen, an antiallergic drug, was found to affect Th1- and Th2-related chemokines [34] by significantly downregulating lipopolysaccharide- (LPS-) induced macrophage-derived chemokines, CXCL-9 (MIG), and CXCL-10 in human leukemia monocytic (THP-1) cells and human primary monocytes. However, other studies on allergic rhinitis using different immunomodulating drugs suggest that an increase in local IFN-*γ* and CXCL10 levels may be associated with symptom relief, suggesting that current information may not be sufficient to completely delineate the exact roles and relationships of these chemokines in the pathogenesis of allergic rhinitis.

### 3.3. Asthma and the CXC Motif Chemokines

Asthma is a complicated and multifaceted pathological disorder with immunological and inflammatory underpinnings. As of 2019, the disease affects around 300 million people worldwide, resulting in over 400,000 fatalities [35]. Widely considered the most common chronic disease among children, asthma is a major cause of disability with a huge social and economic burden in many countries [36]. The characteristic features of this notorious pulmonary disease are inflammation of the airway, remodeling of the airway wall, and hyperresponsiveness of the bronchi [37]. Neutrophils play major roles in asthma, and the levels of neutrophils in the airway submucosa have been positively correlated with asthmatic severity [38]. These polymorphonuclear leukocytes (PMNs) could release reactive oxygen species and lipid mediators as well as tissue-damaging enzymes like neutrophil elastase, which is capable of acting as a secretagogue for goblet cells and potentiating the mucus hypersecretion characteristic of asthma [37, 38]. The CXC chemokine members are potent activators and mobilizers of neutrophils. This is because neutrophils express the CXCR1 and CXCR2 receptors which are, respectively, involved in the activation and recruitment of this leukocyte type [37, 38]. CXCL6 and CXCL8 stimulate the human CXCR1 to a greater extent than CXCL5. CXCR2 not only directs neutrophil migration to inflammatory areas but also inhibits CXCR4-mediated neutrophil retention in the bone marrow by signaling the release of the leukocytes [39]. Both receptors bind CXC chemokine members, namely, CXCL1, CXCL2, CXCL5, CXCL6, CXCL7, and CXCL8 [40]. During the peak of the asthmatic episode, the levels of CXCR2, CXCL5, and CXCL8 were observed to be higher [41]. High amounts of neutrophils and the prototype CXCL chemokine, CXCL8, were detected in the tracheal aspirates of patients suffering from acute severe asthma, and these correlated positively with the severity [42]. Gene transcripts of some of these chemokines (CXCL1, CXCL2, and CXCL5) were found to be upregulated in a mouse model of severe asthma [43]. Mast cells are also implicated in inflammatory processes, such as those associated with asthma. Mast cells in humans express several chemokine receptors, including the CXC motif chemokine receptors CXCR1, CXCR2, and CXCR3, which, respectively, are cognate receptors for CXC pattern ligands such as CXCL8, and CXCL10. CXCR3 is the putative receptor for the three IFN-inducible chemokines, CXCL9, CXCL10, and CXCL11. This receptor is expressed by activated and regulatory T cells, memory T cells, innate lymphoid cells, dendritic cells, and certain B cell subsets. Interactions of these chemokines with their receptors are involved in orchestrating lung mast cell chemotaxis [12, 44]. CXCL10 has been implicated in the migration of mast cells to airway smooth muscle (ASM) in asthmatics [45]. CXCR3 ligands have the ability to recruit mast cells or T lymphocytes to the ASM, where they regulate ASM activities in asthmatic patients [46]. CXCR1 expression on neutrophils may be down-regulated once these leucocyte types have completed their effector roles at the site of inflammation and are ready to migrate across the endothelium, or when inflammatory substances stimulate them [47]. CXCR1 expression on invading neutrophils from asthma or COPD patients, for example, is reduced [12, 48].

#### 3.3.1. Therapeutic Prospects of Antagonism at the CXCL/CXCRs Axis in Asthma

Even though eosinophils are the dominant leukocytes in chronic asthma pathophysiology, there is evidence for considerable involvement of neutrophils as well. CXCL8 levels were found to be elevated in patients' induced sputum [49], fuelling speculations about the potential therapeutic benefit of CXCR inhibition in severe asthma. While CXCL1, CXCL2, and CXCL5 gene transcripts were shown to be elevated in a mouse model of severe asthma, treatment with Ruxolitinib, an anti-inflammatory drug and powerful inhibitor of janus kinase 1/2 (JAK1/2), caused the upward spiraling to be reversed [43]. In an animal model of allergic asthma, tofacitinib, another janus kinase (JAK) inhibitor and immunosuppressant authorized for the treatment of rheumatoid arthritis, was found to lower CXCL1 expression while enhancing immunotherapy efficacy [41, 50]. Also, a dual CXCR1/CXCR2 antagonist, Navarixin (SCH- 527123, MK-7123), was found to reduce circulating and airway neutrophil levels in allergen-induced and severe neutrophilic asthmatic subjects in phase 2 clinical trials (NCT00688467 and NCT00632502), but the improvement in respiratory parameters was not significant [51–53]. The promising profile of AZD5069, a CXCR2 inhibitor, in a chronic obstructive pulmonary disease (COPD) model was not replicated in asthma either, with only a decrease in neutrophil counts in patients' sputum but no improvement in overall clinical outcome [41].

However, CXCR2-dependent promotion of type 2 inflammations and accumulation of key inflammatory players such as neutrophils and eosinophils were attenuated in a mouse model of asthma exacerbation by silencing CXCL3 or CXCL5 via RNA interference, resulting in a reduction in airway hyperreactivity and mucus hypersecretion in asthmatic animals [54]. Lucaks et al. [55] reported the modulation of cytokine profile and Th2-type allergen responses as well as suppression of inflammation and airway hyperactivity in a mouse model of allergen-induced asthma by treatment with a specific CXCR4 inhibitor, plerixafor (AMD3100). Further evidence for the contribution of CXCR4 and its ligand, CXCL12, to the pathophysiology of allergic asthma was demonstrated in ovalbumin-sensitized mice challenged with methacholine. AMD3100 treatment alleviated asthmatic phenotypes such as airway responsiveness and influx of inflammatory cells, which was positively correlated with suppression of the immune response associated with Th17 and Tc17 cells [56].

### 3.4. Acute Lung Injury (ALI)/Acute Respiratory Distress Syndrome (ARDS) and the CXC Motif Chemokines

Acute lung injury (ALI) and its more severe form, acute respiratory distress syndrome (ARDS), are characterized by bilateral pulmonary infiltrations and oxygen therapy-resistant hypoxemia as a result of substantial impairment of alveolar function [57]. Though the etiology of ALI is complex, it is known to involve an inflammatory insult that increases lung permeability and potentiates pulmonary damage with ALI/ARDS-like clinical manifestations [11, 57]. The abundance of neutrophil infiltrations into the pulmonary airspaces as a result of chemoattractant activation by residents' lung cells underscores the role of inflammation in the ALI pathogenesis, and this correlates well with mortality [58]. In the presence of certain chemokines and inflammatory mediators, the shapes and functions of neutrophils are affected, causing them to release toxic intermediates that eventually damage the endothelium and epithelium in the course of migration across these cellular barriers. The damaged epithelium has a reduced capacity for active fluid transport out of the airspaces and surfactant generation, adding to the severity of ALI-related problems such as pulmonary edema and impaired lung compliance [11, 57, 58]. Surfactants and glucocorticoid-based medications have been used in the treatment of ALI/ARDS with mixed results. Only ventilation with decreased tidal volumes produced moderate restoration/protection of the alveolar epithelial integrity, reduced pulmonary edema, and improved overall clinical outcomes [41, 59].

It is known that ELR + chemokines are responsible for neutrophil mobilization from vessels and sequestration in the injured lung and that decreased levels of these granulocytes could attenuate lung vascular permeability and injury indices [60]. They play key roles in ALI/ARDS, as confirmed by evidence from clinical studies. Levels of CXCL1, CXCL5, and CXCL8 are relatively higher in the bronchoalveolar lavage fluid of ALI patients and may correlate with clinical outcomes [11, 60]. Increased CXCL1 levels have also been reported in acute pancreatitis-associated ALI/ARDS, which accounts for 50%–90% of all deaths from pancreatitis [61]. CXCL1, CXCL5, and CXCL8 levels in the plasma and BAL were observed to be elevated in patients at risk for or with ARDS, and a similar finding was made in the case of severe acute pancreatitis when they were found to be strong predictors of disease severity [11, 62]. Macrophages play a key role in ALI. The inflammatory response in ALI/ARDS involves macrophages which secrete CXCL8 to orchestrate the neutrophil recruitment cascade via its receptor, CXCR1 [63]. Following experimentally induced ALI in rodents, there was a marked elevation in the level of CXCL8, a strong chemotactic factor for neutrophils, in the bronchoalveolar lavage fluid. The monoclonal antibody against the chemokine prevented the increase, and this was accompanied by the alleviation of the ALI phenotype [58, 63]. CXCL12 is crucially involved in homing progenitor cells in the bone marrow and also mobilizing the cells into peripheral tissues in the event of injury or stress. Hence, a possible pharmacological role in ALI/ARDS has been proposed for the CXCL12–CXCR4/CXCR7 axis [64].

#### 3.4.1. Therapeutic Prospects of CXCL/CXCRs Axis Antagonism in ALI/ARDS

Blocking the ELR + chemokine's effector receptor, CXCR2, could reduce neutrophil migration into the lungs significantly. For example, in lipopolysaccharide- (LPS-) induced acute lung damage, polymorphonuclear leukocyte migration into the lung of CXCR2-/- mice was significantly reduced [41, 65]. Treatment with a CXCR2 antagonist, antileukinate (Ac-RRWWCR-NH(2)), or anti-CXCL-1 neutralizing antibody reduces inflammatory responses and protects mice from acute pancreatitis-associated ALI or bleomycin-induced acute lung injury [66]. CXCR2 deficiency may protect against septic damage by reducing inflammatory events and enhancing CXCL10 expression [67]. Treatment with antileukinate also disrupted CXCR2 signaling via CXCL1/CXCL2, reversing neutrophil influx and suppressing inflammatory lung injury in a rat hemorrhage/sepsis model of acute lung injury [68]. However, inhibiting angiogenic chemokines alone may not completely prevent neutrophil trafficking, implying that additional sophisticated mechanisms may be at work during immunological activation [63, 68].

CXCR4, which, along with CXCR7, is a cognate receptor for CXCL12, is another intriguing CXCR receptor target. Plerixafor (AMD3100), a nonpeptide antagonist that inhibits the CXCR4/CXCL12 axis, has been shown to promote organ healing by reducing tissue inflammation and stimulating progenitor cell migration to the injury site [69]. In neonatal rats subjected to hyperoxia-induced lung injury (HILI), CXCR4 antagonism was found to decrease pulmonary inflammation and improve alveolarization and lung vascular structure [69]. Preventive and therapeutic treatment with ACT-1004-1239, a CXCR7 antagonist, corrected ALI-associated aberrant breathing patterns and lung vascular damage in a rodent model of LPS-induced ALI/ARDS and reduced immune cell infiltrations of the bronchoalveolar region [64]. The phase 1 clinical trial (NCT03869320) of the promising drug has just been successfully completed [70]. Another study showed that inhibiting CXCR4 and CXCR7 with AMD3100 and CCX771, a powerful, selective CXCR7 antagonist, resulted in decreased inflammatory mediator migration and that the observed anti-inflammatory impact is dependent on a functional adenosine A2B-receptor on hematopoietic cells [71].

### 3.5. Chronic Obstructive Pulmonary Disease (COPD) and the CXC Motif Chemokines

Chronic obstructive pulmonary disease (COPD) is the umbrella term for two lung diseases: chronic bronchitis and emphysema, which affect 10% of adults and are more common in smokers [72]. COPD is the most common chronic inflammatory lung disease, along with asthma [9, 72]. Although different from one another, chronic bronchitis and emphysema present similar symptoms and often occur simultaneously in individuals.

#### 3.5.1. Chronic Bronchitis

In chronic bronchitis, the hair-like projections or cilia that border the bronchioles and serve as a “cushioning carpet” are damaged. This causes the airways to become restricted and irritated, making breathing difficult.

#### 3.5.2. Emphysema

In emphysema, there is an excruciating reduction in the oxygen supply to the blood because the alveoli (air sacs) are enlarged and damaged. The pathological condition is characterized by the disintegration of the alveolar wall as a result of connective tissue deterioration, particularly elastin, and apoptotic processes in the alveolar cells (type 1 pneumocytes) responsible for gas exchange [73].

In COPD, infection, epithelial damage, and inflammation propel the recruitment of neutrophils from the systemic circulation into the airways, causing a vast increase of neutrophils in lung secretions [74]. Exposure to cigarette smoke triggers an inflammatory cascade that activates epithelial cells and macrophages in the respiratory tract. As a result, a large number of chemokines are released, causing inflammatory cells to migrate into the airways [9, 74]. Attempts have been made to elucidate the clear-cut link and contributions of the CXC motif chemokines, CXCL8-12, and cognate receptors, CXCR1-3, in COPD pathogenesis [75]. It has long been known that CXCL8 is crucially involved in COPD and that during exacerbations, the sputum level of this chemokine ligand correlates positively with the neutrophil number. In a similar vein, there are correlations between sputum CXCL8 levels and the neutrophil-associated proinflammatory enzyme, myeloperoxidase in the blood [76]. The CXCL8-CXCR1/2 axis has been recommended as a feasible therapeutic target based on the function of CXCL8 in COPD pathogenesis, and complete neutralization of CXCL8 receptors could be a promising treatment approach for COPD [75]. This proposition was backed up by findings from a phase II clinical trial that showed that a CXCR2 inhibitor, MK-7123, could reduce inflammation and delay the onset of the first exacerbation [77]. CXCL9, CXCL10, and CXCL11 have a common receptor, CXCR3, which binds with differential affinity. The chemokine ligands are produced mainly in the alveolar macrophages and were found to be increased in the sputum of COPD patients with a positive correlation to the number of neutrophils [78]. T cells expressing the common receptor, CXCR3, were detected in larger proportions in COPD patients with substantially impaired lung function. This could point to the role of these chemokines in T-cell recruitment and immune-mediated lung tissue injury [79].

#### 3.5.3. Therapeutic Prospects of CXCL/CXCRs Axis Antagonism in COPD

In COPD patients, IL-10-dependent inhibition of CXCL8 (Il-8) and proinflammatory cytokines (TNF-*α*, IL-1b, IL-6) may be beneficial [49]. CXCL8 is thought to be a biomarker of COPD severity, whereas CXCL10 could be a marker of genetic vulnerability to the disease, with single-nucleotide polymorphisms (SNPs) in certain gene promoters linked to greater or decreased susceptibility to the disease [75]. The effects of CXCL8 could be modulated via its cognate receptors, CXCR1 and CXCR2, which respond to ligand binding by activating neutrophils and inducing chemotactic responses [80]. In light of this, small molecular inhibitors of CXCR2 have been developed that produce partial or complete blocking of neutrophil chemotactic response to IL-8 [81] and have been shown to reduce neutrophil airway inflammation [9]. Two of these inhibitors, SB225002, a strong nonpeptide CXCR2 inhibitor, and SCH527126, which apparently inhibits neutrophils from increasing in the sputum of healthy people after exposure to ozone, are known [80]. In a clinical trial (NCT00551811) with healthy individuals, SB-656933, a CXCR2 selective antagonist, reportedly reduced the expression of CD11b on neutrophil membranes, a cell surface receptor that acts as a marker of neutrophil migration to infection/inflammatory sites [82]. Theinhibition of ozone-induced neutrophilia was connected to this impact [83]. In another study simulating acute cigarette smoke exposure in rats, the infiltration of neutrophils into the lungs of animals was decreased by SB-332235, another CXCR2 antagonist capable of preventing CXCL8 action [84]. Also, following a 6-month long treatment of COPD patients with Navarixin (SCH 527123, MK-7123), there was an improvement in the forced expiratory volume in 1 s (*FEV1*) and a reduction in both exacerbation occurrence time and inflammation [77]. However, due to some reported adverse occurrences including dose-related decreases in absolute neutrophil count, the phase 2 clinical trial (NCT01006616) was discontinued [85]. It is worth noting that some of these chemokines/chemokine receptors are involved in critical physiological functions like immunological defense and antitumor activity; thus, caution should be exercised while inhibiting or blocking them [80]. Also, targeting these chemokines/chemokines receptors in COPD and other inflammatory diseases of the airways is further complicated by the considerable redundancy or overabundance in the chemokine network [86].

### 3.6. Pulmonary Fibrosis and the CXC Motif Chemokines

Pulmonary fibrosis (PF) is a chronic and progressive lung disease characterized by abnormal inflammatory wound healing and excessive deposition of extracellular matrix (ECM) [87]. Studies indicate that CXC chemokines and CXCRs play a role in PF. CXCR4/CXCL12 axis which is involved in the control of lung fibroblast activity has been shown to be required for fibrocyte participation in the pathogenesis of lung fibrosis [88, 89]. CXCL12 acts through CXCR4, a receptor with a role in many biological processes including bone marrow- (BM-) derived stem cell activation and mobilization [90]. Fukushima et al. [87] identified RNA-binding protein 7 (RBM7) and CXCL12 as important components of fibrosis development as both are enhanced in the fibrotic phase. RBM7 orchestrates a cascade of apoptotic events in the lung epithelium as a result of nuclear degradation of NEAT1 noncoding RNA (ncRNA). These events cause the production of CXCL12 and the eventual recruitment of profibrotic segregated-nucleus-containing atypical monocytes (SatMs) to initiate pulmonary fibrosis distinct from the conventional growth factors-driven fibrosis pathway [91]. Also, the potential beneficial role of chemokines which predominate in Th1 cells like CXCR3 in limiting fibrosis was highlighted. This is in contrast with chemokines expressed by Th2 cells like CXCR4, which favor the fibroproliferation process over repair [92, 93]. Mice deficient in CXCR3, which is the receptor for IFN-*γ*-inducible chemokines (CXCL9, CXCL10, and CXCL11), were found to show reduced CXCL10 expression following lung injury with increased mortality and relative susceptibility to interstitial fibrosis [92]. Exogenous or endogenous IFN-augmentation, on the other hand, could improve the fibrosis phenotype in CXCR3-deficient mice [92]. This underscores the relevance of angiogenic and angiostatic CXC chemokines in the pathophysiology of pulmonary fibrosis. The levels of angiogenic (CXCL1, CXCL2, CXCL3, CXCL5, CXCL6, CXCL7, CXCL8, and CXCL12) and angiostatic (CXCL4, CXCL9, CXCL10, CXCL11, and CXCL14) chemokines have been found to be positively and negatively correlated, respectively, with fibrosis [94, 95]. Furthermore, depletion of angiogenic CXC chemokines or supplementation with the angiostatic ones resulted in the mitigation of fibrosis due to a reduction in pulmonary angiogenesis [94, 95].

#### 3.6.1. Therapeutic Prospects of CXCL/CXCRs Axis Antagonism in Pulmonary Fibrosis

Treatment of bleomycin-induced fibrotic rats with the CXCR4 antagonist, AMD3100, reduced CXCL12 levels in bronchoalveolar lavage (BAL) fluids, resulting in a reduction in pulmonary fibrocytes and an overall improvement in pulmonary fibrosis phenotypes [96]. The authors concluded that restricting the recruitment of fibrocytes to the injured lung by blocking the CXCL12/CXCR4 axis with a CXCR4 antagonist could prevent fibrotic events [96]. More evidence for AMD3100's known effects on the CXCR4/CXCL12 axis and pulmonary fibrosis was provided by Li et al. [88]. In human lung fibroblasts (HLFs), the CXCR4 antagonist reduced CXCL12 and CXCR4 expression, as well as HLF proliferation and CXCR expression potentiation in response to exogenous CXCL12 stimulation [88]. These events were also associated with a decrease in the severity of the pathophysiology of pulmonary fibrosis. In addition, AD-114, a single domain antibody that is highly specific for CXCR4, suppressed collagen release by IPF fibroblasts, reduced fibrocyte accumulation in fibrotic murine lungs, and improved the amount of pulmonary damage in mice with experimentally induced idiopathic pulmonary fibrosis (IPF) [97].

Chow et al. [98] have proposed that in pulmonary fibrosis, the damaged lung releases CXCL12, which stimulates the proliferation of local hepatic stellate cells (HSCs) and elevation of CXCR4 expression, ultimately leading to collagen 1 synthesis. Another inhibitor of the CXCL12/CXCR4 axis, AMD070, could improve mortality associated with bleomycin-induced pulmonary injury, but this was linked with the dampening of early inflammatory or injurious events as the overall impact on extracellular matrix deposition was negligible [98].

### 3.7. Bronchiectasis and the CXC Motif Chemokines

Bronchiectasis is a chronic respiratory disease characterized by persistent and permanent enlargement and thickening of the bronchial tubes with consequent mucus build-up in the lung, compromised mucus clearance/airway blockages, breathing difficulties, and recurring lung infections. The condition is incurable but manageable, and its prevalence rises with age and the female gender [99]. Bronchiectasis is divided into two types: cystic fibrosis and noncystic fibrosis bronchiectasis. The latter, known as idiopathic bronchiectasis, has largely unknown causes even though previous severe pulmonary-damaging infections, some genetic, autoimmune, and inflammatory diseases including primary ciliary dyskinesia, rheumatoid arthritis, and Crohn's disease, as well as allergic reactions, have all been implicated. Bronchiectasis is characterized by an atrocious cycle of infection, inflammation, and tissue damage [100]. The pathophysiology of bronchiectasis centers on the development of neutrophilic inflammation in the airways [101], as well as CD4+ T cells and CD68+ macrophage infiltration [102]. CXCL-8 levels were discovered to be high in both CF and non-CF bronchiectasis and comparable to COPD in neutrophilic bronchiectasis [103, 104]. The level of the chemokine in sputum was directly correlated with neutrophils, the severity of bronchiectasis, and the frequency of exacerbations [104]. In a prospective study to evaluate the pattern and role of inflammatory mediators in bronchiectasis-related small airway dysfunction (SAD) in patients with humoral immunodeficiency, severe neutrophilic inflammation was evident in induced sputum and bronchial inflammation was paralleled by proinflammatory mediators CXCL-8 and IL-1*β* [105].

#### 3.7.1. Therapeutic Prospects of CXCL/CXCRs Axis Antagonism in Bronchiectasis

In bronchiectasis, targeting excessive neutrophilic inflammation could be a promising strategy. It was suggested that intervention aimed at facilitating airway clearance of CXCL-8 and/or serine proteases may reduce airway inflammation in CF bronchiectasis [100]. Absolute sputum neutrophil count was shown to be significantly lower in a randomized placebo-controlled investigation of the effects of AZD5069, a CXCR2 antagonist, in people with bronchiectasis. However, this did not translate to improved clinical outcomes as there were no indications of relevant beneficial effects on sputum weight or lung function [106]. The involvement of CXCR1 in CXCL-8 binding and neutrophil trafficking prompted the analysis of the gene variant, +2607 G/C, in non-CF bronchiectasis and normal control individuals, and the discovery of a similar pattern of distribution in both conditions indicates that the chemokine receptor might not be involved [107].

### 3.8. Lung Cancer and the CXC Motif Chemokines

Lung and pulmonary cancers continue to be the leading causes of cancer-related fatalities in both male and female humans [108]. The two main types of lung cancer are small cell lung cancer (SCLC) and non-small-cell lung cancer (NSCLC), with the latter being the most common (up to 85 percent). NSCLC is divided into three subtypes: adenocarcinoma, squamous cell carcinoma, and giant cell carcinoma, which are distinguished by their histology and treatment modalities [109]. The level of CXCL1 protein in human adenocarcinoma was found to be significantly higher than in adjacent normal lung tissue, and similar CXCL1 level elevation in blood was found to be positively correlated with clinical stage, including advanced tumor growth and metastasis, which was associated with poor overall survival [110]. It has previously been hypothesized that lung cancer cells express and release large levels of CXCL1 and that knocking down the angiogenic CXC chemokine suppresses tumor growth in animals by inhibiting infiltration of tumor-associated neutrophils into tumor tissues from peripheral blood [111].

CXCL5, a member of the proangiogenic subgroup of the CXC family, has been found to play a key role in tumor development and metastasis by interacting with its GPCR, CXCR2, and activating both the traditional EGFR and RSK1/2/AKT/ERK pathways, leading to phosphorylation of HSP27 (Heat Shock Protein) [112]. CXCL5 overexpression in lung cancer is associated with poor differentiation, advanced disease stage, and poor overall survival, whereas CXCL5 deficiency is linked to reduced angiogenesis, delayed tumor progression, and metastatic tendencies [109].

Similarly, CXCL8 is an angiogenic CXCL-type chemokine implicated in tumor microenvironment neovascularization, and a strong positive correlation was found between the chemokine and CXCL1 [113]. CXCL8 promotes the activation of matrix metalloproteinase (MMP) and enhances tumor growth and metastasis [114]. High levels of CXCL8 (and CXCL1) in lung adenocarcinoma are linked to increased cancer risk and a poor disease prognosis, whereas low expression of CXCL1, CXCL7, and CXCL8 is associated with a better prognosis [114, 115]. Furthermore, CXCR1/2 expression was found to be reduced in lung cancer patients, and these receptors are linked to neutrophil and macrophage infiltration [114]. Because CXCL8 is not expressed in mice, CXCL2 has been utilized to assess CXCL8-related signaling pathways since they share the CXCR2 receptor [116]. CXCR2 is the sole or exclusive receptor for all ELR + CXC chemokines and has been implicated as a mediator of their angiogenic activity [117].

Although most studies link CXCR2 to carcinogenesis, one study found that a single-nucleotide polymorphism (SNP) in CXCR2's 3' untranslated region (UTR) induces greater CXCL receptor expression but is associated with a lower risk of lung cancer [118]. CXCR2 has also been revealed to be capable of playing a tumor suppressive role since the receptor can mediate p53-dependent senescence in the lung [119, 120]. It is possible that CXCR2's role in tumor suppression varies depending on the stage of the tumor. Targeting CXCR2 in cancer management is a promising adventure but might just not be totally efficient or curative due to the complexity of the development and metabolic outlook of cancer. Hence, multiple treatment regimens were suggested [120]. The general belief is that the angiostatic ELR-CXC chemokines, CXCL4, CXCL9, CXCL10, and CXCL11, unlike their ELR + counterparts, tend to oppose angiogenesis and endothelial cell growth [109, 121]. Together with their receptor, CXCR3, they might be involved in tumor elimination by inhibiting angiogenesis and recruiting immune and inflammatory cells to engage tumors in mortal combat. Increased CXCR3 expression in NSCLC clinical samples correlated with immune cell infiltration and extended survival [122, 123]. In support of this, administration of the hematopoietic growth factor, IL-7, caused increased CXCR3 expression on tumor-associated T cells and immune cell infiltration and decreased tumor burden [122]. Furthermore, reduced CXCL10 levels are associated with poor prognosis, and restoration of CXCL10 in the human adenocarcinoma cell line A549 led to inhibition of tumorigenesis without increased leukocyte infiltration [122]. CXCL10 and CXCL11 may also be useful diagnostic biomarkers in early-stage NSCLC, while CXCL8 levels may be a useful predictor of future lung cancer risk [121].

While the CXCL9/10/11/CXCR3 axis is more generally associated with antitumorigenesis, studies of its participation in cancer growth and metastasis have been published [124]. Their involvement has been connected in one case to the presence of tumor CXCR3 receptors that can promote (CXCR3-A) or inhibit (CXCR3-B) tumor growth [125]. Enhancement of paracrine CXCR3 ligands (CXCL9, CXCL-10, and CXCL-11) in the tumor microenvironment and deactivation of CXCR3 expression on cancer cells could be antitumorigenic by recruiting activated natural killer cells and T lymphocytes with potent antitumor effects in various lung cancer models [124, 125]. Some of the reasons implicated in the differential involvement of CXCR3 in lung cancer include differences in damaged tissue, the level of inflammation, and the types of immune cells produced as a result of cancer types and organs involved [124].

The CXCL13 : CXCR5 axis might be involved in the promotion of lung cancer, and the B cell chemoattractant chemokine (CXCL13) is elevated in NSCLC patients, suggesting that it could be used as a predictor of the risk of early-stage lung adenocarcinoma [126]. Mice lacking CXCL13 and CXCR5 had impaired tumor formation after being exposed to environmental carcinogens such as polycyclic aromatic hydrocarbons (PAH), whereas lung tumors developed in mice after being exposed to the PAH benzo(a)pyrene (Ba[a]P) had elevated CXCL13 levels, highlighting the importance of the chemokine axis in lung tumorigenesis [127]. In most lung cancer types, the CXCL12/CXCR4 axis is tumor-promoting. High CXCR4 expression in tumor cells promotes invasiveness and metastasis, which is exacerbated by high CXCL12 expression in lymph nodes but can be prevented by inhibiting the receptor [128]. Antagonism of CXCR4 could potentially boost the chemosensitivity of tumor cells [128, 129]. It was reported that CXCL13 markedly increased the production of the cytokine secreted by macrophages, secreted phosphoprotein-1 (SPPI, or osteopontin in humans) in mice. The collaboration of CXCL13-CXCR5-SPPI may promote the EMT phenotype in tumors from B[a]P-treated mice, causing mesenchymal markers like vimentin and N-cadherin to be upregulated while E-cadherin is downregulated [126, 130]. Following the stimulation of the NF-*κ*B transcription factor, CXCL13 and its cognate receptor, CXCR5, promoted lung cancer cell migration by upregulating vascular cell adhesion molecule-1 (VCAM-1) expression in an event that involved multiple signaling pathways, including protein kinase C-(PKC), phospholipase C-(PLC), and c-Src [131]. In NSCLC patients, CXCR5 expression was higher in carcinomas compared with noncancerous tissues [130, 132]. Increased CXCL17 expression in NSCLC has also been demonstrated and could be linked to possible protection of cancer cells against apoptosis and promotion of metastatic tendencies, leading to poor survival [132].

#### 3.8.1. Therapeutic Prospects of CXCL/CXCRs Axis Antagonism in Lung Cancer

As previously reported, CXCR2 expression was found to be higher in both human lung adenocarcinoma and lung squamous cell carcinoma, and this was linked to patient prognosis. SB225002, a specific CXCR2 inhibitor, promoted apoptosis and senescence in lung cancer cells while decreasing EMT and proliferation [133]. The inhibitor reduced neutrophil infiltration and tumor growth in a rodent model of lung cancer while also enhancing antitumor T-cell activity and cisplatin's anticancer potency [133]. This is in support of a previous study showing that in Lewis lung cancer orthotopic and heterotopic tumor model systems, rendering CXCR2 ineffective via gene knock-out or with a specific neutralizing antibody lowered tumor development and spreading potential [117, 134].

In another study, suppression of the CXCR1/2 axes with a mutant CXCL8 analog and CXCR1/2 antagonist, CXCL8(3-72)K11R/G31P (G31P), in H460 and A549 lung cancer cell lines caused dose-dependent repression of cell proliferation and migration and increased apoptosis of cancer cells. *In vivo*, tumor development, metastatic events, and angiogenesis were all dramatically reduced following CXCR1/2 suppression with G31P in a study with an orthotopic xenograft mouse model of human lung cancer [135]. Downregulation of vascular endothelial growth factor (VEGF) and nuclear factor-kappa B-p65 (NF-*κ*B-p65), as well as reduced phosphorylation of extracellular signal-regulated protein kinase (ERK) 1/2 and protein kinase B (AKT), may be the underlying molecular mechanisms [135]. Navarixin (MK-7123) has also been demonstrated to be beneficial in the treatment of NSCLC. The CXCR2/CXCR1 antagonist was used in conjunction with Pembrolizumab (MK-3475) in a phase 2 clinical trial (NCT03473925) to assess the safety and efficacy of both medicines in adults with solid malignancies, including NSCLC [136]. Another study showed that the CXCR4 inhibitor, AMD3100, improved the antimetastatic potential of conventional anticancer drug (etoposide and cisplatin) combinations in an animal model of small cell lung cancer, SCLC [137]. In addition, a recent study involving tumors from NSCLC patients and lung cancer cell lines found that inhibiting CXCR4 with peptide R significantly reduced the dissemination of metastasis-initiating cells and their immunosuppressive tendencies [138].

### 3.9. Pneumonia and the CXC Motif Chemokines

Pneumonia is a deadly, acute lung disease characterized by inflammatory-driven fluid/pus-filled alveoli, resulting in breathing difficulty and reduced oxygen availability. The clinical condition is usually caused by bacterial (*Streptococcus pneumoniae*), viral (Respiratory syncytial virus, influenza A/B), or fungal (Coccidioides) respiratory infections, or a combination of these [139].

#### 3.9.1. Viral Pneumonia and Therapeutic Prospects of CXCRs Axis Antagonism

Chemokines, in general, aid in the fight against viral infections by recruiting immune cells to the infection site, enhancing their cytotoxic activities, and promoting the production of antiviral mediators [140]. Chemokines, on the other hand, have been used by viruses to evade the immune system in some cases [141]. CXCL10 and other interferon-inducible chemokines may play a role in the host antiviral response in respiratory tract infections by encouraging viral clearance prior to adaptive immune system activation [142]. Interferon-inducible chemokines, including CXCL10, could be vital to the host antiviral response in respiratory tract infections by promoting viral elimination prior to the activation of the adaptive immune system [142]. Levels of inflammatory chemokines like CXCL8, CXCL9, and CXCL10 were found to be elevated in some diseased states and might correlate with disease severity [140]. Immune cell recruitment by inflammatory chemokines and associated cytotoxic responses sometimes exaggerates the accompanying inflammation and results in tissue damage. In this backdrop, these chemokines may be beneficial or harmful in the event of viral infections of the pulmonary tissues, and inhibiting them has been proposed as a viable treatment strategy to combat some viruses [140, 143]. While investigating the involvement of the CXCL1-3/CXCR2 axis in dsRNA (Poly IC)-induced viral infection of the lung, it was discovered that the expression of CXCR2 and its ligands, CXCL1 and CXCL2/3, tallied with the recruitment of neutrophils to the lung [144]. The CXCL1-3/CXCR2 biological axis is a critical facilitator of lung injury in this setting since neutrophil mobilization and pulmonary injury were reduced upon treatment of mice with anti-CXCR2 neutralizing antibody following prior exposure to dsRNA [144]. The CXCL5/CXCR2 axis might also be involved in the regulation of leukocyte mobilization after pulmonary influenza infection [145]. Reparixin, a CXCR2 inhibitor with a promising profile in the treatment of severe COVID-19 pneumonia, has completed a phase 3 clinical trial (NCT04878055) in adult patients with the disease [146]. Furthermore, CXCL5 inhibition causes infection-fighting macrophages and monocytes to express CXCL13, a B cell chemokine, resulting in B cell accumulation, immune response enhancement, and the orchestration of induced bronchus-associated lymphoid.

#### 3.9.2. Bacterial Pneumonia, Bacterial Coinfection, and Therapeutic Prospects of CXCL/CXCRs Axis Antagonism

*Streptococcus pneumoniae* is one of the most common bacteria implicated in the development of bacterial pneumonia (pneumococcus). The role of CXCL1 in the pathophysiology of bacterial-induced pneumonia was demonstrated using CXCL1-/- mice [147]. Toll-like receptor- (TLR-) and NF-*κ*B-associated increases in CXCL1 were protective, as the chemokine augmented neutrophil influx to mitigate bacterial growth and dissemination in the pulmonary tissue, resulting in lower mortality. Exogenous administration of CXCL2 and CXCL5, which also function through CXR1/CXR12, could thus reverse the effect of CXCL1 knock-out and boost survival rates [147]. Similarly, CXCL2 expression was induced following *Klebsiella pneumoniae* exposure, and inhibition of the chemokine was associated with the decreased neutrophil influx into the pulmonary airways, bacterial clearance, and early survival [148]. Mice lacking the murine chloride channel accessory 3, mCLCA3, a murine ortholog of the human hCLCA1 implicated in inflammatory pulmonary disease, showed reduced neutrophilic infiltration after *Staphylococcus aureus*-induced pneumonia, which was accompanied by lower mRNA and protein levels of CXCL-1 (a murine CXCL-8 homolog), although there was no effect on mucus cell metaplasia or overall clinical outcome [149]. Coinfections are common in pulmonary respiratory diseases and secondary bacterial infections after severe viral infections constitute a huge cause for concern due to the associated high mortality, as was the case of the Spanish Flu [150]. In such cases, the synthesis of factors involved in neutrophil chemotaxes, such as CXCL1 and CXCL2, may be impaired [151]. Treatment with the CXR1/2 antagonist, DF2162, significantly reduced the inflammatory response, increased bacterial burden and CXCL1/2 levels, lung damage, and morbidity associated with secondary pneumococcal infection in IAV-treated murine hosts without compromising the animals' defense mechanisms against infection [152]. Blocking the CXCL8/CXCR1/2 axis with CXCL8 (3–72)G31P (pG31P), a CXCR1/2 antagonist, effectively mitigated LPS-induced pneumonia in mice by reducing the production of inflammatory cytokines such as CXCL8, TNF-*α*, and IL-1 and limiting neutrophil infiltration into the pulmonary parenchyma [153].

In light of the foregoing arguments, it makes sense to believe that chemokine and chemokine/receptor axis modulation in the event of bacterial pneumonia has a greater impact on the extent of inflammatory cell mobilization and subsequent inflammatory events than on other pneumonia phenotypes or overall clinical outcomes.

### 3.10. Tuberculosis and the CXC Motif Chemokines

Tuberculosis (TB) is a pulmonary disease caused by the bacteria *Mycobacterium tuberculosis* (Mtb). Symptoms include a chronic cough with blood-tinged mucus, weight loss, fever, and night sweats. Smoking and an HIV/AIDS infection could make *tuberculosis* worse [154]. With nearly 10 million active infections and 1.5 million deaths estimated in 2020, the disease only trailed COVID-19 as the largest cause of infection-related deaths [155]. Although the roles of the CXC chemokines in TB have not been fully characterized, there is enough evidence for their involvement in the pathophysiology of the infectious disease. The neutrophil-mediator chemokine, CXCL8, was found elevated in TB sputum samples and at sites for positive tuberculin skin reactions while the serum levels decreased following antibiotic treatment in a corresponding manner to neutrophil levels [156]. The reduced CXCL8 following treatment could be related to either disease improvement or a reduction in neutrophil accumulation [156]. CXCL8 levels, on the other hand, were found to be substantially linked to the severity and pathogenesis of tuberculosis-induced acute respiratory distress syndrome (ARDS) [157].

A recent prospective case-control study of children with and without *tuberculosis* found that baseline levels of CXCL1 and CXCL10 were exclusively higher in the infected people and decreased after anti-TB treatment. Further statistical analyses demonstrated that the chemokines have over 80% specificity and sensitivity, making them an accurate biomarker for active *tuberculosis* diagnosis [158]. The CXCL10 (IP-10) level was found to be decreased in the course of a 6-month-long anti-TB therapy and could be a useful indicator in identifying and monitoring the progression of pulmonary TB as well as the efficiency of therapeutic interventions [159]. A single-nucleotide polymorphism (SNP) in the CXCL-10 promoter (135G > A), located a few base pairs upstream of the NF-*κ*B binding site, reportedly enhances susceptibility to TB [160]. Furthermore, CXCL5 and its receptor, CXCR2, are elevated in *M. tuberculosis* infection, although this could be linked to destructive rather than beneficial neutrophilic inflammation, emphasizing the importance of excessive polymorphonuclear leukocyte (PMN) infiltration in pulmonary injury. Following high-dose *M. tuberculosis* infection, the survival of both CXCR2 and CXCL5 knock-out mice was found to be enhanced compared to the respective wild types due to impaired PMN recruitment [161].

In addition, CXCL13, the B cell chemoattractant, and its cognate receptor, CXCR5, could be involved in TB pathophysiology due to their importance to B cell biology. The chemokine level is elevated in Mtb-infected mice where it regulates CXCR5+T-cell localization to the lung parenchyma, phagocytosis, and bacterial cell growth. On the other hand, CXCR5 is necessary to maintain Mtb-specific CD4+ T cells upon chronic infection [162]. Mice deficient in CXCL13 and CXCR5 appear to be more susceptible to Mtb infection. The CXCL13/CXCR5 axis has thus been proposed to play a nonredundant role in Mtb infection since deficiency in any other chemokine/receptor axis does not produce this effect [156, 162].

#### 3.10.1. Therapeutic Prospects of CXCL/CXCRs Axis Antagonism in Tuberculosis

In pulmonary *tuberculosis*, there is a paucity of scientific reports on the effects of small molecular inhibitors and/or specific antibodies on CXC chemokine ligands and their receptors. It has been postulated that in active TB, CXCL10 undergoes posttranslational processing to an antagonist form, possibly as a result of DPP4-dependent N-terminal truncation, which could play a regulatory role and open up potential therapeutic opportunities. Such DPP4-induced homing of Th1 T cells to the TB-infected lung could prevent harmful overinflammation but could also slow down T-cell elimination of intracellular infection [163]. Apart from the CXCL10/CXCR3 chemotactic axis, CXCR2, TNF-a, and IL-1 receptors on granulocytes are frequently deregulated following initial exposure to mycobacterial pathogens, possibly to limit excessive inflammation by inflammatory cells at the site of exposure [164].

## 4. Conclusion

A plethora of evidence from numerous studies utilizing experimental approaches such as small molecule receptor antagonists/agonists, neutralizing antibodies of CXC chemokine receptors, and animal models deficient in or overexpressing CXC receptors and/or their ligands supports the involvement of CXC chemokines and their cognate receptors in the pathophysiology of lung diseases. Chemokines have important roles in inflammation and immune surveillance, and most lung illnesses are inflammatory and/or immunological in nature. As a result, this subfamily of chemokines has an impact on a variety of lung disorders, ranging from allergic rhinitis to *tuberculosis* and lung cancer, while impacting critical signaling pathways such as NF-*κ*B, EGFR, and RSK1/2/AKT/ERK. It is established that the exquisite and specific pharmacological inhibition/manipulation of CXC chemokines and receptors could have therapeutic value in the context of lung disorders. This is critical as there are multiple interacting cytokines and chemokines with similar functional effects implicated in the etiology of pulmonary diseases and which may mask the intervention's effects. Also, due to the crucial physiological activities of the CXC chemokines, inhibiting these immune mediators could result in complications or adverse effects, which could overshadow any potential favorable impact. When CXC chemokines/receptor manipulations alone do not suffice to treat lung illnesses, they could be useful as an adjuvant therapy. A better understanding of the biology of CXCLs/CXCRs in lung diseases would allow the circumvention of noticeable barriers to the effective utilization of these immune and inflammatory mediators in the treatment of lung disease.

## Data Availability

No data were used in the study.

## Abbreviations

AKT/PKB: Protein kinase B
ALI/ARDS: Acute lung injury/acute respiratory distress syndrome
cAMP: Cyclic adenosine monophosphate
COPD: Chronic obstructive pulmonary disease
CXCL: C-X-C motif chemokine ligand
CXCR: CXC motif chemokine receptor
DPP4: Dipeptidyl peptidase 4
dsRNA: Double-stranded ribonucleic acid
EGFR: Epidermal growth factor receptor
ERK: Extracellular signal-regulated protein kinase
G-CSF: Granulocyte colony-stimulating factor
GPCRs: G protein-coupled receptors
HILI: Hyperoxia-induced lung injury
IFN-*γ*: Interferon-gamma
ILDs: Interstitial lung diseases
IPF: Idiopathic pulmonary fibrosis
I-TAC: IFN-inducible T-cell alpha chemoattractant
JAK: Janus kinase
mCLCA3: Murine chloride channel accessory 3
NF-*κ*B: Nuclear factor-kappa B
NSCLC: Non-small-cell lung cancer
PI3K: Phosphoinositide 3-kinase
PIP2: Phosphatidylinositol 4,5-bisphosphate
PIP3: Phosphatidylinositol (3,4,5)-trisphosphate
PMN: Polymorphonuclear leukocyte
RBM7: RNA-binding protein 7
RSK1/2: Ribosomal s6 kinase 1/2
SatMs: Segregated-nucleus-containing atypical monocytes
SNP: Single-nucleotide polymorphism
TLR: Toll-like receptor
TNF-*α*: Tumor necrosis factor-alpha.
